# The accuracy of presepsin (sCD14-ST) for the diagnosis of sepsis in adults: a meta-analysis

**DOI:** 10.1186/s13054-015-1032-4

**Published:** 2015-09-11

**Authors:** Xin Zhang, Dan Liu, You-Ning Liu, Rui Wang, Li-Xin Xie

**Affiliations:** Department of Pulmonary & Critical Care Medicine, General Hospital of Chinese People’s Liberation Army, 28 Fuxing Road, Beijing, 100853 PR China; Department of Clinical Pharmacology, General Hospital of Chinese People’s Liberation Army, 28 Fuxing Road, Beijing, 100853 PR China; Medical School, Nankai University, 94 Weijin Road, Tianjin, 300071 PR China; Department of Respiratory Medicine, Tianjing Medical University General Hospital, Tianjin, 300070 PR China

## Abstract

**Introduction:**

The early diagnosis of sepsis remains a challenge. Recently, soluble cluster of differentiation 14 subtype (sCD14-ST), also known as presepsin, has been identified as a potential biomarker of sepsis. We performed a meta-analysis to assess the diagnostic accuracy of presepsin for sepsis in patients with systemic inflammation.

**Methods:**

We systematically searched the PubMed, Embase, Web of Knowledge and Cochrane databases. Studies were included if they assessed the diagnostic accuracy of presepsin for sepsis in adult patients with systemic inflammatory response syndrome (SIRS). Furthermore, a 2 × 2 contingency table was constructed based on these results. Two authors independently judged the studies and extracted the data. The diagnostic accuracy of presepsin in sepsis was calculated using a bivariate meta-analysis model. The Q-test and *I*^2^ index were used to test the heterogeneity.

**Results:**

Eight studies involving a total of 1,815 patients were included in the present study. The pooled sensitivity, specificity, diagnostic odds ratio, positive likelihood ratio and negative likelihood ratio were 0.86 (95 % CI: 0.79-0.91), 0.78 (95 % CI: 0.68-0.85), 22 (95 % CI: 10–48), 3.8 (95 % CI: 2.6-5.7), and 0.18 (95 % CI: 0.11-0.28), respectively. The area under the summary receiver operator characteristic curve was 0.89 (95 % CI: 0.86–0.92). Meta-regression analysis revealed that consecutive patient selection, sample size and setting significantly accounted for the heterogeneity of sensitivity.

**Conclusions:**

Our findings suggest that presepsin exhibits very good diagnostic accuracy (AUC=0.89) for the diagnosis for sepsis. Nevertheless, an overall assessment of all the clinical indexes for sepsis diagnosis and continual re-evaluation of presepsin during the course of the disease are needed.

## Introduction

Sepsis is a life-threatening condition that arises when the body’s response to an infection injures its own tissues and organs [1]. Despite advances in antibiotic therapy and modern life support, the fatality rate of patients with sepsis has remained as high as 30−60 % [2, 3]. Delays in the initiation of antimicrobial treatment are associated with worse prognosis [4, 5], which highlights the importance of timely diagnosis to reduce the morbidity and mortality of sepsis patients.

Currently, the diagnosis of sepsis is based on the presence of systemic inflammatory response syndrome (SIRS) criteria in the presence of a known infection. However, non-infectious SIRS associated with acute tissue injury and innate immune activation can induce clinical syndromes analogous to sepsis, including multiple trauma, pancreatitis, burns, and autoimmune diseases. Blood culture is the gold standard for identifying infectious conditions, but this approach is of limited use for the early detection of a bloodstream infection due to the duration of time required to obtain positive cultures, exclude specimen contamination and identify colonization [6]. Biomarkers, which were recently introduced among the inflammatory variables in the diagnostic criteria for sepsis, could contribute to the prompt identification of sepsis. However, no ideal biomarker has yet been identified with sufficient clinical sensitivity or specificity for the diagnosis of sepsis [7]. An ideal biomarker is still needed to accurately differentiate sepsis from non-infectious SIRS in a timely and economic manner.

Cluster of differentiation 14 (CD14) is a multifunctional glycoprotein expressed mainly on the membrane surface of monocytes/macrophages. CD14 is also minimally distributed on the cell surface of neutrophils (mCD14) and serves as a specific receptor for complexes of lipopolysaccharides (LPSs) and LPS-binding proteins (LPBs). The LPS-LBP-CD14 complex is released into the circulation by shedding CD14 from the cell membrane, yielding soluble CD14 (sCD14), which is also directly secreted by hepatocytes [8, 9]. During inflammation, plasma protease activity generates (sCD14) fragments. The 64-amino acid N-terminal fragments constitute the sCD14 subtype (sCD14-ST), which has recently been renamed presepsin [10]. Presepsin has recently been reported to increase in response to the severity of bacterial infections. The plasma levels of presepsin specifically increase during sepsis, and less intensively, during SIRS. An increasing number of studies have demonstrated the ability of presepsin to serve as a valuable marker in sepsis diagnosis [11–18]. In light of the above findings, we aimed to assess the value of presepsin for the diagnosis of sepsis by performing meta-analysis of relevant studies of diagnostic test accuracy. To the best of our knowledge, this approach has not been previously performed.

## Methods

### Search strategy and selection criteria

We systematically searched studies in the PubMed, Embase, Web of Knowledge and the Cochrane Library databases. The search terms were as follows: (“presepsin” or “soluble CD14 subtype” or “sCD14-ST”) AND (sepsis OR “bacterial infection” OR “systemic inflammatory response syndrome” OR “SIRS”). No language restriction or publication date restrictions were applied. We further reviewed the reference lists of the selected articles to obtain potentially relevant articles.

Eligible studies were required to include a well-defined reference standard for the patients involved. For the included studies, the sepsis group was defined according to the criteria of the American College of Chest Physicians/Society of Critical Care Medicine [19, 20], whereas the other patients were required to meet the criteria for SIRS. Additionally, the studies included data on the diagnostic accuracy of presepsin for sepsis in adult patients (>18 years old) with SIRS. Furthermore, a 2 × 2 contingency table was constructed based on the results. For studies providing multiple presepsin cutoff values for diagnostic accuracy, the data providing the maximum overall accuracy were chosen. For studies that evaluated the diagnostic accuracy of presepsin levels at multiple time points, we chose the data based on the initial presepsin level. Reviews, letters, commentaries, correspondence, case reports, conference abstracts, expert opinions, editorials and animal experiments were excluded. Articles involving paediatric patients were also excluded. Two investigators (Zhang X and Liu D) independently performed the search strategy and evaluated the studies. Any disagreement was resolved by a third opinion.

### Data extraction and quality assessment

The following descriptive data were extracted from the original studies: the name of the first author, publication year, country of origin, study design, clinical setting, severity of sepsis, control patients, sample size, prevalence of sepsis, the percentage of male patients, average age, cutoff points, and the true positive (TP), false positive (FP), false negative (FN), and true negative (TN) rates, sensitivity (SEN) and specificity (SPE) of the data. We contacted the corresponding authors if the necessary data were not presented or required clarification. We used a revised tool for the Quality Assessment of Diagnostic Accuracy Studies (QUADAS) checklist [21] to assess the quality of the included studies. Furthermore, the studies were grouped according to Sackett and Haynes’ classification of diagnostic studies [22]. Utilizing this classification, phase 1 studies were those that compared the difference in test results between patients with the target disorder and healthy individuals. Phase 2 studies were those that examined how the index test differentiated between patients with and without the target disorder. Phase 3 studies were those that assessed the test’s real-life performance in patients in whom the disorder was suspected.

### Statistical analysis

All statistical analyses were performed using the MIDAS module for STATA software, version 12.0 (Stata Corporation, College Station, TX, USA). *P* values less than 0.05 were considered statistically significant. A bivariate random effects regression model [23] was used to calculate the pooled SEN, SPE, diagnostic odds ratio (DOR), positive likelihood ratio (PLR), and negative likelihood ratio (NLR). We also constructed a summary receiver operator characteristic (SROC) curve by plotting the individual and summary points of SEN and SPE to assess overall diagnostic accuracy [24]. Fagan’s nomogram was used to calculate the post-test probability (PTP) [25]. The *Q* test and *I*^2^ index were calculated to assess between-study heterogeneity [26, 27]. *I*^2^ greater than 50 % was considered sustainable heterogeneity among studies.

In addition to the proportion of heterogeneity that was likely due to the threshold effect, univariate meta-regression analysis and subgroup analysis were performed to explore the sources of potential heterogeneity in SEN and SPE. The covariates included the following: prevalence (prevalence <50 % or ≥50 %), sample size (sample size <100 or ≥100), blinding (whether sepsis patients were diagnosed without knowledge of the results of the presepsin level), setting (whether the study was performed in the emergency department (ED) or intensive care unit (ICU)), study design (whether patients were recruited consecutively), and comorbidities (whether the studies excluded patients who had comorbidities that were likely to influence presepsin levels). Subgroup analysis was restricted to ED patients. Publication bias was tested by Deek’s funnel plot.

## Results

**Fig. 1:**
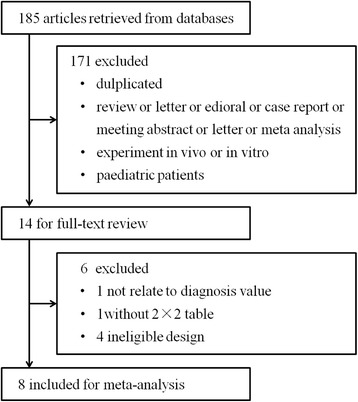
Flow chart of study selection

Our database search retrieved 185 articles, 172 of which were eliminated for various reasons based on the title and abstract, leaving 13 studies that were scrutinized in a full-text review. Among the 13 studies, one study investigated the prognostic value of presepsin in sepsis, one could not be used to reconstruct the 2 × 2 table, and three were performed using an ineligible design (i.e., they evaluated the diagnostic accuracy of presepsin based on samples that were collected at multiple time points but not every individual sample). In total, eight studies fulfilled our eligibility criteria and were included in the final analysis (Fig. 1). We did not identify any additional relevant articles in the bibliographies of the original articles.

### Characteristics of the included studies

The included studies were published between 2012 and 2014. Four studies [11, 14, 16, 18] were conducted in Europe, and four [12, 13, 15, 17] were conducted in Asia. A total of 1,815 patients were included, and SIRS criteria were fulfilled in 1,690 patients, including 1,165 patients with septis and 525 patients with non-infectious SIRS. Four studies recruited a group of well-matched (by age and sex) patients without SIRS (60 patients in Behnes [11], 25 patients in Kweon [12], 100 patients in Liu [13], and 70 patients in Vodnik [14]) as controls, and two studies [12, 13] included the control patients as well as the SIRS patients in the non-sepsis group in a 2 × 2 contingency table when analysing the diagnostic accuracy of presepsin for sepsis. The mean age of the patients varied between 54.4 and 79.42 years, and the proportion of male patients ranged from 50.0 to 66.3. The prevalence of sepsis varied from 16.4 % to 79.2 %. The most frequent source of sepsis was pulmonary infection. Six studies [12–14, 16–18] were performed in the ED, one [11] in the ICU, and one [15] in the ED and ICU. All included studies recruited a mix of medical and surgical patients. One study [12] excluded patients with comorbidities (e.g., chronic renal failure or a history of resuscitation and trauma) that could influence presepsin levels. Presepsin levels were measured with a chemiluminescent immunoassay on a PATHFAST immunoanalyzer [28, 29] in all studies. The test threshold ranged from 317 to 729 pg/ml. Details of all eight studies are presented in Table 1. The optimal cutoff point was retrospectively determined based on the ROC curve. The mean cutoff for presepsin in the included studies was 560 pg/ml (IQR 317–729).

**Table 1:** Characteristics of included studies

Author	Year	Country	Study design	Clinical setting	Severity of sepsis	Control patients	Sample size (n)	Prevalence (%)	Male (%)	Mean/medianage (years)	Cutoff (pg/ml)	TP	FP	FN	TN	SEN	SPE
Group 1 studies																	
Kweon [12]	2014	Korea	PR	ED	Sepsis, severe sepsis and septic shock	25 patients without SIRS	118	61.9	50	61.19 (20–91)	430	64	8	9	37	87.7	82.2
Liu [13]	2013	China	PR+CR	ED	Sepsis, severe sepsis and septic shock	100 healthy controls	859	79.2	66.3	79.42 (58–78)	317	481	40	199	239	70.8	85.8
Group 2 studies																	
Vodnik [14]	2013	Serbia	PR	ED	Sepsis, severe sepsis and septic shock	70 healthy controls	60	50	58.3	54.4 ± 15.5	630	30	2	0	28	100	93
Endo [15]	2012	Japan	MPR	ED and ICU	NA	No	185	62.1	59.5	71.8 (17–98)	600	101	13	14	57	87.8	81.4
de Guadiana Romualdo [16]	2014	Spain	PR	ED	NA	No	226	16.4	58.4	67 ± 26	729	30	70	7	119	81.1	63
Ishikura [17]	2014	Japan	PR	ED	Sepsis, severe sepsis and septic shock	No	82	52.4	53.7	67.2 ± 17.3	647	39	9	4	30	93	76.3
Ulla [18]	2013	Italy	MPR	ED	Sepsis and septic shock	No	189	56.1	61.4	64.4 (19–99)	600	84	32	22	51	78.95	61.9
Behnes [11]	2014	Germany	PR	ICU	Severe sepsis and septic shock	60 patients without SIRS	96	84.4	63.1	65.89 (20–88)	530	73	6	8	9	90	60

*PR* prospective recruitment, *MPR* multicentre prospective recruitment, *CR* consecutive recruitment, *ED* emergency department, *NA* not available *TP* true positive, *FP* false positive, *TN* true negative, *FN* false negative, *SIRS* systemic respiratory response syndrome, *SEN* sensitivity, *SPE* specificity

### Study quality and publication bias

**Fig. 2:**
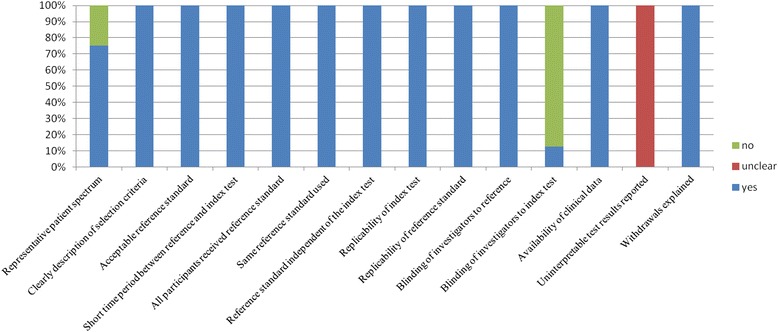
Proportion of Quality Assessment of Diagnostic Accuracy Studies (QUADAS) tool criteria fulfilled by the included studies

**Fig. 3:**
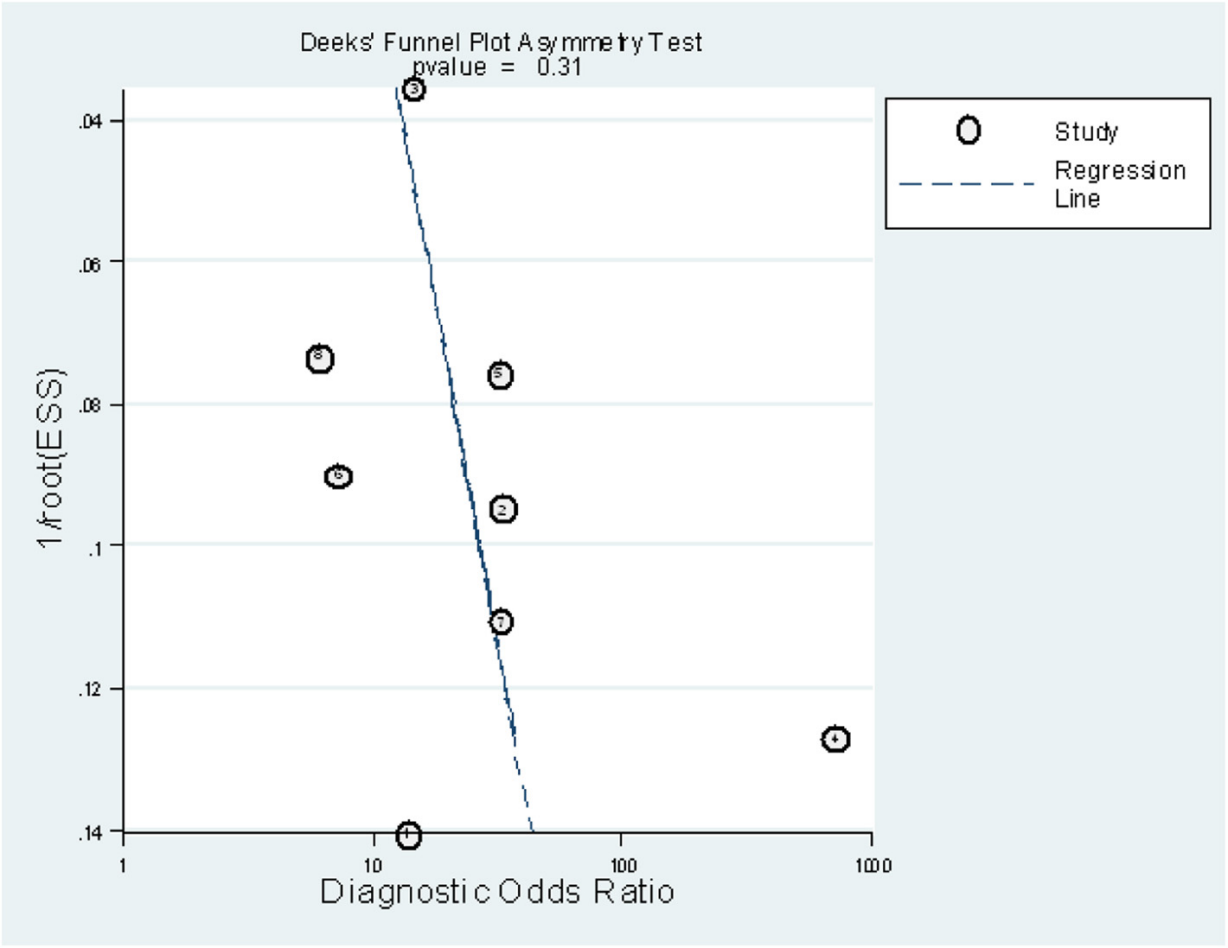
Deek’s funnel plot asymmetry test for publication bias. No publication bias was detected (*p* = 0.31)

Studies were grouped according to Sackett and Haynes’ classification for diagnostic studies: two were phase 2 studies (group 1) [12, 13], and six were phase 3 studies (group 2) [11, 14–18]. All studies included a prospective cohort. Two [15, 18] studies were multicentre trials. One study [13] consecutively enrolled patients. Two studies [12, 13] excluded patients with comorbidities that could influence presepsin levels. The QUADAS checklists are presented in Fig. 2. On average the overall QUADAS scores of all studies met 10 of the 14 criteria, which suggests that the studies were of high quality. Deek’s funnel plot is presented in Fig. 3. No significant publication bias was observed (*p* = 0.31).

### Data synthesis and meta-analysis

**Fig. 4:**
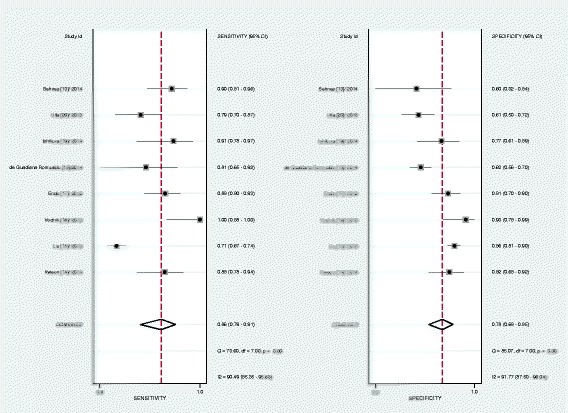
Forrest plot of the sensitivity and specificity of presepsin for the diagnosis of sepsis

**Fig. 5:**
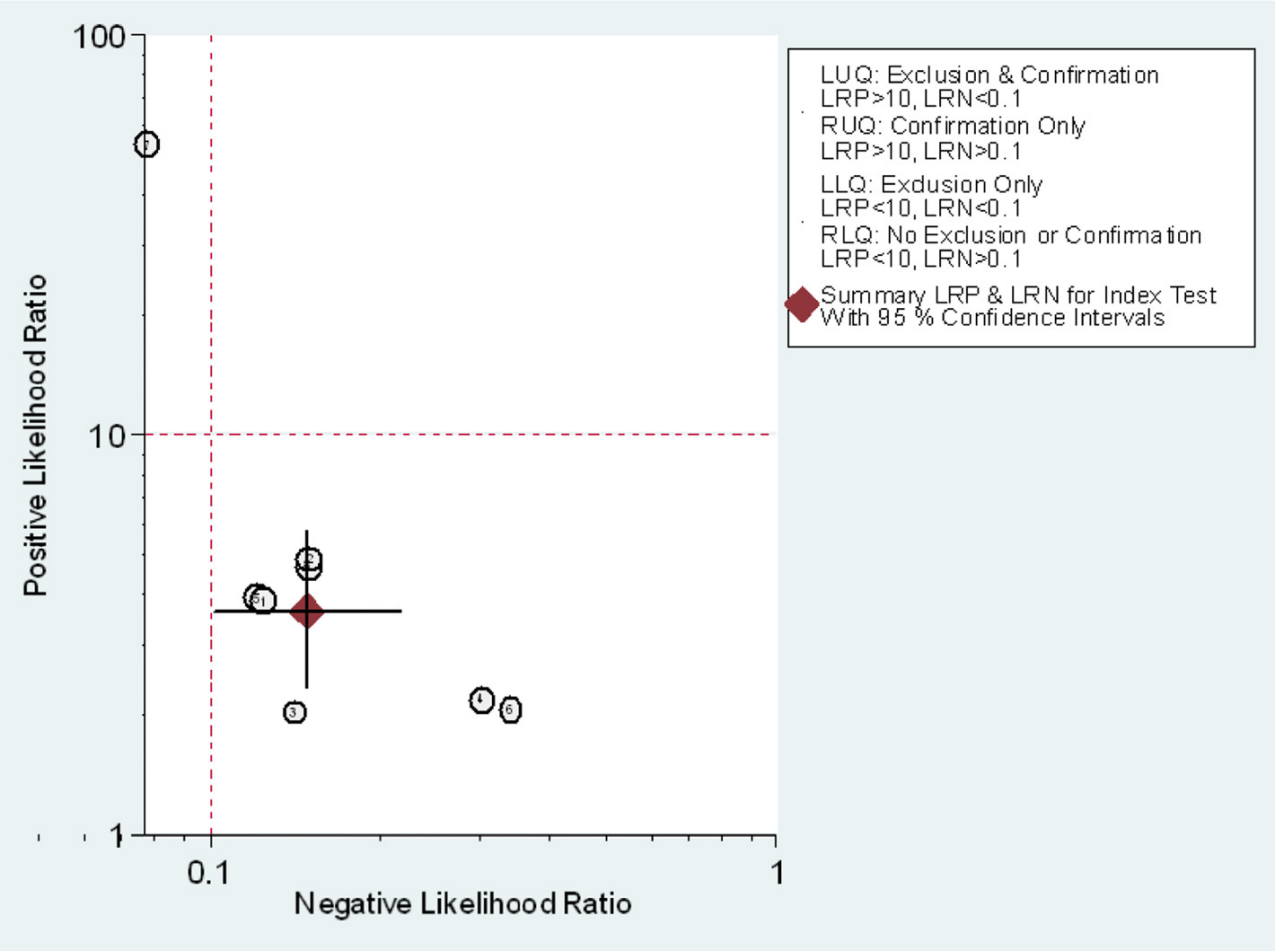
Positive and negative likelihood ratios. The positive likelihood ratio and negative likelihood ratio were 3.8 (95 % CI: 2.6, 5.7), 0.18 (95 % CI: 0.11, 0.28), respectively LUQ left upper quadrant, RUQ right upper quadrant, LLQ left lower quadrant, RLQ right lower quadrant, LRP likelihood ratio positive, LRN likelihood ratio negative

**Fig. 6:**
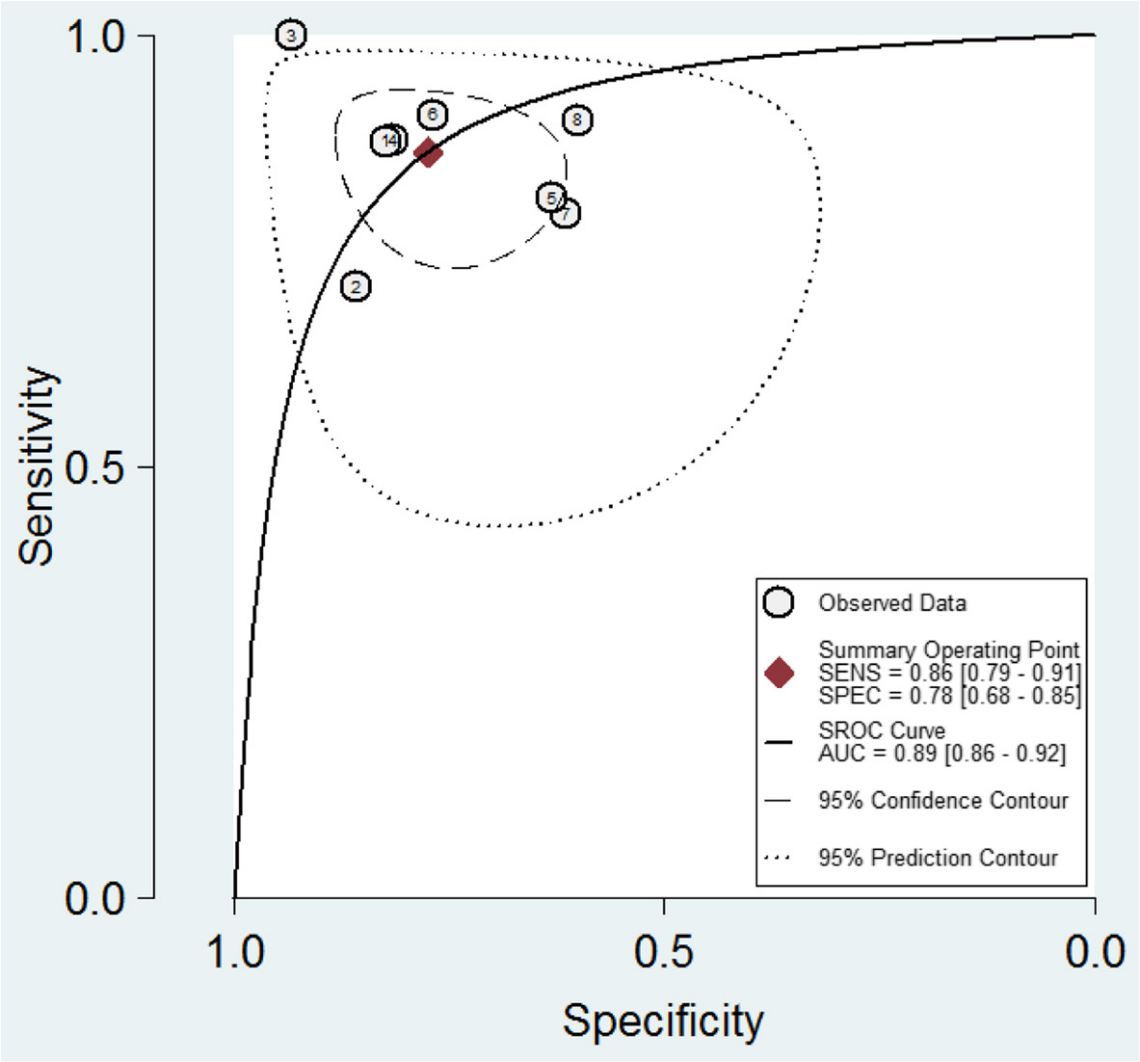
Summary receiver operating characteristic graph of included studies. *SEN* sensitivity, *SPE* specificity

**Fig. 7:**
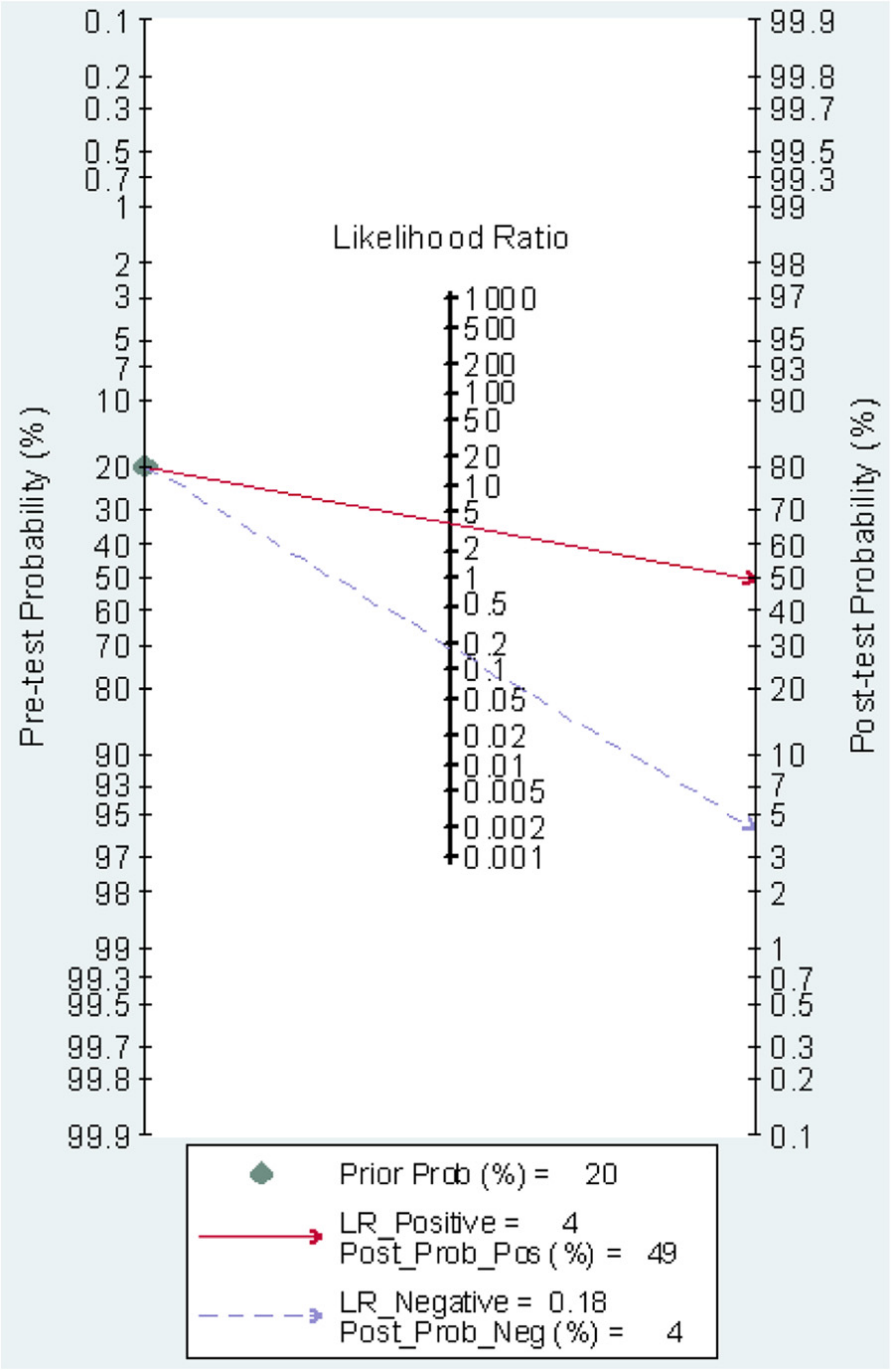
Fagan’s nomogram for calculation of post-test probabilities. Fagan’s nomogram for presepsin illustrates post-test probability with fixed pre-test probability of 20 % for sepsis. *LR* likelihood ratio, *pos* positive, *neg* negative

The pooled SEN and SPE were 0.86 (95 % CI: 0.79, 0.91) and 0.78 (95 % CI: 0.68, 0.85), respectively (Fig. 4). The PLR and NLR were 3.8 (95 % CI: 2.6, 5.7) and 0.18 (95 % CI: 0.11, 0.28), respectively (Fig. 5). The DOR was 22 (95 % CI: 10, 48). The area under the SROC curve was 0.89 (95 % CI: 0.86, 0.92) (Fig. 6). Figure 7 presents Fagan’s nomogram for likelihood ratios, and the results indicate that the use of presepsin in the detection of sepsis increased the post-probability to 48 % when the results were positive and reduced the post-probability to 4 % when the results were negative. The mean cutoff for presepsin in the included studies was 560 pg/ml (IQR 317–729).

Six studies comprising 838 patients were included in group 2 studies (Table 1). We performed a subgroup analysis restricted to this group because the studies were restricted to patients who were most likely to be encountered by clinicians and who were more informative for routine clinical practice. The pooled SEN and SPE were 0.89 (95 % CI: 0.81, 0.94) and 0.75 (95 % CI: 0.64, 0.84), respectively. The PLR and NLR were 3.6 (95 % CI: 2.2, 5.8) and 0.15 (95 % CI: 0.08, 0.29), respectively. The DOR was 24 (95 % CI: 8, 71). The area under the SROC curve was 0.90 (95 % CI: 0.87, 0.92). The *I*^***2***^ test results for the pooled SEN and SPE were 68.22 % and 84.90 %, respectively.

There was substantial heterogeneity among the studies. The *I*^***2***^ test results for the pooled SEN and SPE were 90.49 % and 91.77 %, respectively. The overall *I*^***2***^ values for the bivariate model were 94 % (95 % CI: 85, 99). The proportion of heterogeneity likely caused by the threshold effect was small (0.07), whereas the variations in SEN and SPE were related to differences in the cutoff points for presepsin that were used in the included studies.

**Fig. 8:**
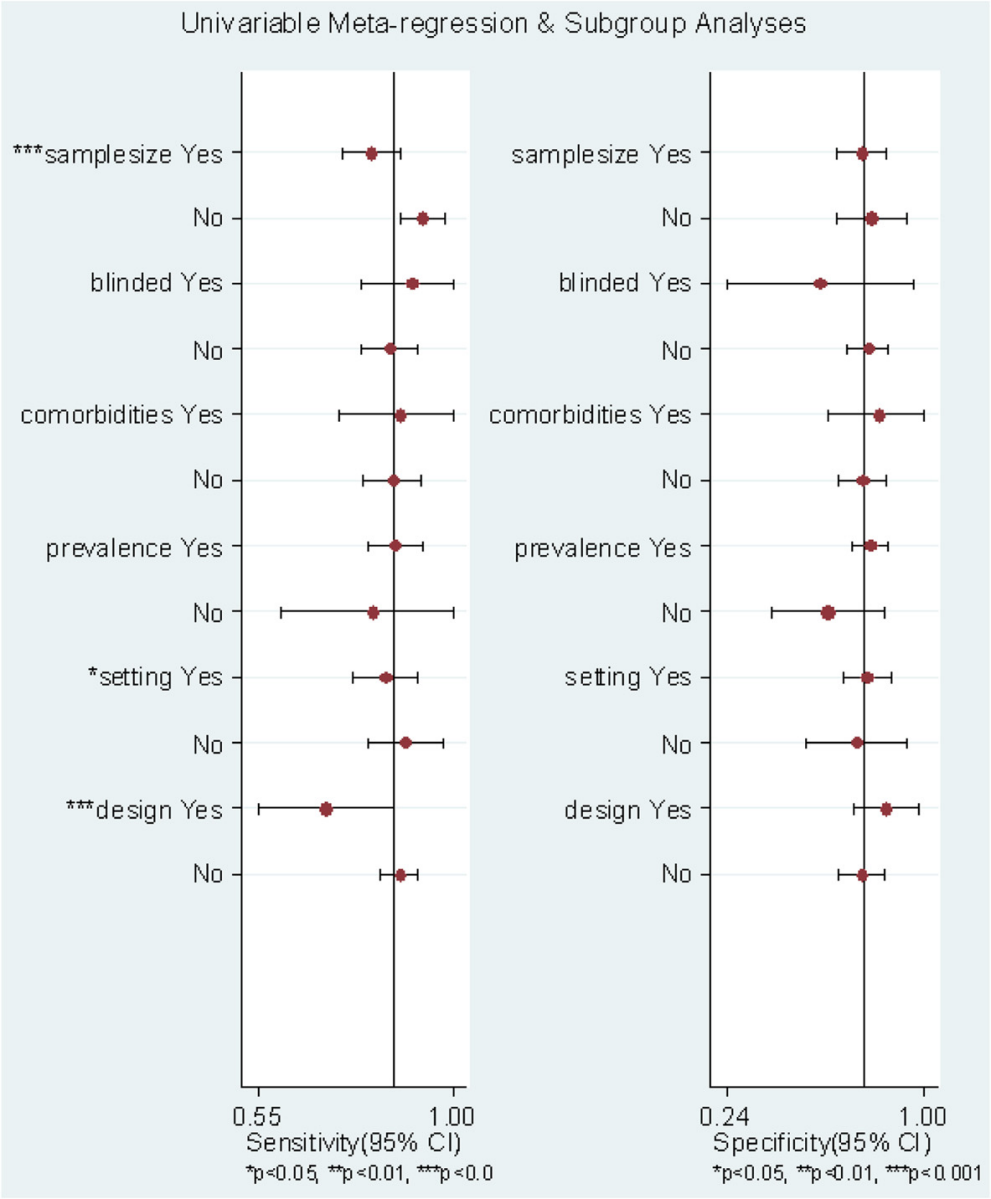
Univariate meta-regression and subgroup analysis

Univariate meta-regression analysis and subgroup analysis were performed to explore the sources of potential heterogeneity in SEN and SPE. Patient blinding, prevalence, setting, consecutive patient recruitment, and sample size were used as covariates. Meta-regression revealed that consecutive recruitment, sample size, and setting significantly accounted for the heterogeneity of sensitivity (Fig. 8). The subgroup analysis restricted to ED patients revealed that the pooled SEN and SPE were 0.85 (95 % CI: 0.77, 0.92) and 0.78 (95 % CI: 0.69, 0.88), respectively.

## Discussion

Sepsis is a common problem in critically ill patients. Early diagnosis and early treatment are essential for the clinical course and the outcome of patients with sepsis. Given that a large proportion of critically ill patients exhibit SIRS, the ability to accurately distinguish between SIRS and sepsis, which is defined as SIRS as a result of bacterial infection, has become one of the holy grails of critical care medicine.

Currently, no recommendation can be given for the use of biomarkers to differentiate sepsis from non-infectious SIRS [30]. Many biomarkers, particularly procalcitonin (PCT) and C-reactive protein (CRP), are widely used to identify sepsis in current clinical practice [31, 32]. In comparison with CRP, PCT seems to be a better marker to differentiate sepsis from non-infectious SIRS. However, two published meta-analyses concluded that the SEN and SPE of PCT varies in the diagnosis of sepsis, leading to questions of the ability of PCT to distinguish sepsis from SIRS [33, 34]. Presepsin was recently identified as a molecule involved in the inflammatory response [35] and represents a promising diagnostic biomarker with high SEN and SPE.

The primary finding of our meta-analysis is that presepsin exhibits very good diagnostic accuracy for distinguishing patients with sepsis from those with systemic inflammatory disease. Specifically, in our primary analysis involving all studies that evaluated presepsin in adults with and without sepsis, the area under the SROC curve was 0.89, which was greater than the results of published meta-analyses of the use of PCT and soluble triggering receptor expressed on myeloid cells-1 (sTREM-1) for the diagnosis of sepsis [33, 34, 36]. The pooled SEN and SPE were 86 % and 78 %, respectively. To our knowledge, presepsin exhibited the highest sensitivity among the proposed biomarkers in differentiating sepsis form other non-infectious SIRS. To date, no biomarkers have exhibited sufficient (greater than 90 %) sensitivity to distinguish sepsis from SIRS in these critically ill adult patients.

The rescue principles indicate that the infection foci of patients with sepsis should be detected within 6 hours, followed by antibiotic treatment within 1 hour after the diagnosis of sepsis [37]. Generally, PCT increases 4 hours after infection, slowly reaching a plateau at 8–24 hours and peaking one day after infection. Compared with PCT, presepsin increases at 2 hours post-infection in the cecal ligation and puncture (CLP) sepsis model and peaks at 3 hours [29]. Presepsin can be detected in the early stage of infection using rapid dosage methods based on chemiluminescence enzyme immunoassay, which are available and permit automated measurements in 1.5 hours [38].

The exclusion of reviews, letters, commentaries, correspondence, case reports, conference abstracts, expert opinions, editorials and reports of animal experiments may have contributed to publication bias. However, we tested for this, and no significant publication bias was observed in our study (Fig. 3).

Marked statistical heterogeneity was present in all analyses, a fact that must not be overlooked in the interpretation of the above findings. We observed significant heterogeneity in SEN and SPE among the studies analysed. Consecutive patient recruitment, sample size, setting and excluded patients substantially affected the SEN of the diagnosis of sepsis in the meta-regression analysis, and none of the variables affected the SPE of diagnosis of bacterial infection in the meta-regression analysis. According to Sackett and Haynes’ classification [22], the index test in group 1 is developed in an ideal situation against a validation set (group 2) in which the performance is tested in a more realistic clinical context. Group 2 studies are the most informative for clinical practice, as they are designed to resemble the real-world setting of routine clinical practice by restriction to patients who are the most likely to be encountered by clinicians. In this meta-analysis, six of the included studies were classified as group 2 studies. Additionally, we performed subgroup analysis restricted to group 2. The pooled sensitivity, DOR and AUC of the six studies (11, 14–18) were similar to corresponding values in the other studies. In particular, the pooled AUC indicated high diagnostic accuracy (AUC ≥0.9) [39]. All of the studies included involved a prospective cohort, and two were multicentre studies, underlining the high quality of these studies. The test results obtained by the prospective recruitment (PR) study design method achieved more realistic results than that obtained by the consecutive recruitment (CR) method. Therefore, satisfactory results could be achieved in the future by implementing more prospective studies.

Likelihood ratios and PTPs are also relevant for clinicians, as they provide information on the likelihood of a patient with a positive or negative test actually exhibiting sepsis. In our study, with a hypothetical pretest probability of 20 % and a PLR of 3.8, detecting presepsin for sepsis diagnosis would raise the PTP to 49 %, with an NLR of 0.18. Detecting presepsin reduced the PTP to 4 % (Fig. 7), demonstrating that the application of the presepsin test was advantageous in the diagnosis of sepsis. Additionally, all included studies recruited a mix of medical and surgical patients. Our findings can therefore be generalized to patients from different countries as well as to different admission categories.

Delayed resuscitation is reportedly significantly associated with a high risk of death [40, 41], and rapidly initiating the appropriate therapeutic interventions upon the patient’s arrival to the ED is critical. Thus, we performed subgroup analysis restricted to ED patients to evaluate the diagnostic accuracy of presepsin. The pooled SEN and SPE were 0.85 (95 % CI: 0.77, 0.92) and 0.78 (95 % CI: 0.69, 0.88), respectively, nearly equal to the overall results.

Several limitations should be considered when interpreting the findings of this meta-analysis. First, despite the extensive literature search, the number of included studies was small; however, the number of patients enrolled was satisfactory (n = 1,815), thereby decreasing type II error. Second, we could not determine the optimized cutoff value because we failed to obtain the raw data to map the ROC curve. In all studies, the optimal cutoff point was retrospectively determined based on the ROC curve. The cutoff points varied greatly among the studies, despite using the same presepsin assay. A reason for this discrepancy may be differences in study design, especially the patient inclusion criteria. Falsely elevated values of presepsin or PCT are observed in conditions of chronic renal failure or a history of resuscitation and trauma. One study [12] excluded patients with these comorbidities, but the others did not. Thus, future research should be designed in consideration of how comorbidities may influence presepsin levels to confirm an optimal cutoff point for clinical use. Third, due to the small number of eligible studies and the lack of necessary data reported in the original publications, we could not specifically analyse patients with different conditions (e.g., different severities of sepsis or different sites of infection) to distinguish the sepsis, nor could we determine the therapeutic decisions in the individual patient. Last, it is possible that presepsin may perform differently in sepsis caused by gram-positive, gram-negative, or fungal pathogens. Hence, both the clinical characteristics of the enrolled patients as well as the local microbiological profile in included studies are likely to affect the value of presepsin in predicting sepsis. However, we were unable to explore this further because the necessary information was largely unavailable in the studies.

## Conclusions

Despite these limitations, our study is the first comprehensive meta-analysis to date that assesses the diagnostic accuracy of presepsin for sepsis in patients with SIRS. The results demonstrated that presepsin may be a reliable biomarker for sepsis because of its good overall diagnostic performance. However, presepsin cannot be recommended as the single definitive test for sepsis diagnosis according to the current data. Clinicians must comprehensively evaluate the condition of each patient and should incorporate an overall assessment of the clinical indexes for sepsis diagnosis rather than focusing only on a single biomarker-based approach. Moreover, continuous re-evaluation during the course of the disease is advisable.

## Key messages

- The present study showed that the pooled sensitivity and specificity of presepsin for sepsis diagnosis was 0.86 and 0.78, respectively. The area under the SROC curve was 0.89
- Presepsin has very good diagnostic accuracy (AUC = 0.89) for the diagnosis for sepsis in patients with SIRS. However, it cannot be recommended as the single definitive test for sepsis diagnosis according to current data

## Abbreviations

CR: consecutive recruitment
CRP: C-reactive protein
DOR: diagnostic odds ratio
ED: emergency department
FN: false negative
FP: false positive
ICU: intensive care unit
NLR: negative likelihood ratio
PCT: procalcitonin
PLR: positive likelihood ratio
PR: prospective recruitment
PTP: post-test probability
sCD14-ST: soluble CD14-subtype
SEN: sensitivity
SIRS: systemic inflammatory response syndrome
SPE: specificity
TN: true negative
TP: true positive
